# Heterologous Overexpression of Poplar *SnRK2* Genes Enhanced Salt Stress Tolerance in *Arabidopsis thaliana*

**DOI:** 10.3389/fpls.2016.00612

**Published:** 2016-05-09

**Authors:** Xueqing Song, Xiang Yu, Chiaki Hori, Taku Demura, Misato Ohtani, Qiang Zhuge

**Affiliations:** ^1^Key Laboratory of Forest Genetics and Biotechnology, Co-Innovation Center for Sustainable Forestry in Southern China, Ministry of Education, Nanjing Forestry UniversityNanjing, China; ^2^Biomass Engineering Program Cooperation Division, RIKEN Center for Sustainable Resource ScienceYokohama, Japan; ^3^Graduate School of Biological Sciences, Nara Institute of Science and TechnologyIkoma, Japan

**Keywords:** *SnRK2*, overexpression, salt stress, transport, metabolism, salt tolerance, poplar

## Abstract

Subfamily 2 of SNF1-related protein kinase (SnRK2) plays important roles in plant abiotic stress responses as a global positive regulator of abscisic acid signaling. In the genome of the model tree *Populus trichocarpa*, 12 *SnRK2* genes have been identified, and some are upregulated by abiotic stresses. In this study, we heterologously overexpressed the *PtSnRK2* genes in *Arabidopsis thaliana* and found that overexpression of *PtSnRK2.5* and *PtSnRK2.7* genes enhanced stress tolerance. In the *PtSnRK2.5* and *PtSnRK2.7* overexpressors, chlorophyll content, and root elongation were maintained under salt stress conditions, leading to higher survival rates under salt stress compared with those in the wild type. Transcriptomic analysis revealed that *PtSnRK2.7* overexpression affected stress-related metabolic genes, including lipid metabolism and flavonoid metabolism, even under normal growth conditions. However, the stress response genes reported to be upregulated in Arabidopsis *SRK2C/SnRK2.6* and wheat *SnRK2.8* overexpressors were not changed by *PtSnRK2.7* overexpression. Furthermore, *PtSnRK2.7* overexpression widely and largely influenced the transcriptome in response to salt stress; genes related to transport activity, including anion transport-related genes, were characteristically upregulated, and a variety of metabolic genes were specifically downregulated. We also found that the salt stress response genes were greatly upregulated in the *PtSnRK2.7* overexpressor. Taken together, poplar subclass 2 *PtSnRK2* genes can modulate salt stress tolerance in Arabidopsis, through the activation of cellular signaling pathways in a different manner from that by herbal subclass 2 *SnRK2* genes.

## Introduction

Plants face various environmental stresses including drought, high salinity, and extreme temperatures. Such adverse circumstances can often lead to severe agricultural and industrial losses, so it is important to understand the molecular and physiological mechanisms that plants use to cope with abiotic stresses for further stable production of crops and biomass feedstock. Many studies have indicated that regulatory factors of protein phosphorylation play essential roles in response to environmental stimuli (Sopory and Munshi, 1998; Umezawa et al., 2013). One of the well-characterized protein kinases involved in stress responses is the group of sucrose non-fermenting 1 (SNF1)-related protein kinases (SnRKs; Halford and Hey, 2009). SnRKs are grouped into three subfamilies, SnRK1, SnRK2, and SnRK3 (Halford and Hey, 2009), and recent studies have indicated pivotal roles of plant-specific subgroups of SnRK2 and SnRK3 in the link between abiotic stress and abscisic acid (ABA) signaling to regulate metabolic pathways (Hrabak et al., 2003; Halford and Hey, 2009). Increasing evidence shows that SnRK2 proteins function as positive regulators of ABA signaling for stress responses, as well as development, in plants (Umezawa et al., 2013). In *Arabidopsis thaliana* (Arabidopsis) and rice, the SnRK2 family includes 10 members, such as SRK2A-SRK2J or SnRK2.1–2.10 in Arabidopsis and SAPK1-10 in rice (Yoshida et al., 2002; Hrabak et al., 2003; Kobayashi et al., 2004), and they are further classified into three subclasses based on their domain structures (Kobayashi et al., 2004). Most SnRK2 proteins are activated by abiotic stresses, while the members of subclasses 2 and 3 are also activated by ABA (Boudsocq et al., 2004, 2007; Kobayashi et al., 2004). In the current model, ABA-induced activation is largely explained by the interaction between SnRK2s and protein phosphatase type 2C (PP2C) proteins in the ABA signaling pathway (Leung et al., 1994, 1997; Meyer et al., 1994; Saez et al., 2004; Nishimura et al., 2007; Umezawa et al., 2009; Cutler et al., 2010; Ng et al., 2014). In the absence of ABA, group A PP2Cs physically bind to SnRK2s to dephosphorylate SnRK2s, resulting in the inhibition of ABA signal transduction, while in the presence of ABA, SnRK2 will be released from such inhibitory regulation by PP2C, because the soluble ABA receptor PYR/PYL/RCAR inhibits PP2C activity (Umezawa et al., 2009; Vlad et al., 2009).

In Arabidopsis, detailed analyses of subclass 2 (SRK2F/SnRK2.7 and SRK2C/SnRK2.8) and subclass 3 (SRK2D/SnRK2.2, SRK2I/SnRK2.3, and SRK2E/SnRK2.6) have revealed their redundant functions in ABA signaling for abiotic stress responses and developmental controls (Yoshida et al., 2002; Fujii et al., 2007; Fujii and Zhu, 2009; Fujita et al., 2009; Nakashima et al., 2009; Mizoguchi et al., 2010). Importantly, overexpression of *SnRK2* genes resulted in enhanced abiotic stress tolerance in Arabidopsis (AtSRK2C/SnRK2.8, Umezawa et al., 2004; TaSnRK2.3, TaSnRK2.4, TaSnRK2.7, and TaSnRK2.8, Mao et al., 2010; Zhang et al., 2010, 2011; Tian et al., 2013) and in rice (SAPK4; Diédhiou et al., 2008). Overexpression of *SnRK2* genes in Arabidopsis induced the upregulation of several important stress responsive genes, including RD29A and DREB1A/CBF3, and ABA biosynthetic genes, such as ABA1, under normal conditions (Umezawa et al., 2004; Zhang et al., 2011), suggesting that early and quick stress responses supported by the expression of such key genes may enhance stress tolerance in Arabidopsis. The rice *SAPK4* overexpressor showed increased salt tolerance, and major aspects of its tolerance were explained by changes in the expression of genes related to ion homeostasis and oxidative stress responses (Diédhiou et al., 2008). In the cases of SnRK2 overexpressors, the results clearly indicated that SnRK2 can function in abiotic stress responses in plant cells, through the modulation of stress response-related gene expression.

Comparative genomics studies have demonstrated that the core components of ABA signaling, PYR/PYL/RCAR, SnRK2, and PP2C, are well-conserved in land plant species (Umezawa et al., 2010), suggesting the evolutionary conservation of a molecular system involving these proteins in land plants. Indeed, the conserved molecular characteristics of SnRK2, such as transcriptional induction by abiotic stresses and activation by stress and/or ABA, have been reported for SnRK2 genes not only in Arabidopsis and rice, but also in other crop plants: maize (Huai et al., 2008; Vilela et al., 2012) and wheat (Holappa and Walker-Simmons, 1995; Gómez-Cadenas et al., 1999; Mao et al., 2010; Zhang et al., 2010, 2011; Tian et al., 2013). Additionally, we recently confirmed the physical interaction between SnRK2 and PP2C in a model tree *Populus trichocarpa* (poplar), and proposed the possibility that a similar molecular module containing SnRK2 and PP2C is involved in the ABA signaling pathway in trees (Song et al., 2015). However, information about the molecular functions of the poplar SnRK2 proteins is still limited. Recently, we evaluated the transcriptional regulation of *PtSnRK2* genes and found that some of them are upregulated by abiotic stresses in organ-specific manners, suggesting the involvement of PtSnRK2 in ABA-dependent and/or ABA-independent regulation of stress responses (Yu et al., unpublished data.).

In this study, to obtain further clues as to the molecular functions of PtSnRK2 proteins, we heterologously overexpressed the *PtSnRK2* genes in Arabidopsis. Our data indicated that poplar subclass II *PtSnRK2* genes can enhance the salt stress tolerance of Arabidopsis, and that poplar *PtSnRK2* overexpression would activate cellular signaling and stress response pathways in Arabidopsis in a different manner than that by the herbal subclass II *SnRK2* genes.

## Materials and methods

### Plant materials and growth conditions

Young shoots of black cottonwood, *P. trichocarpa* Torr. & A. Gray (poplar), grown in 15-cm-high plant pots, were used for the cloning of *PtSnRK2* cDNA. For the overexpression analysis, *A. thaliana* (Arabidopsis) plants (Columbia strain) were used. The growth conditions were described in Ohtani et al. (2011) for poplar and in Ohtani et al. (2013) for Arabidopsis.

### Plasmid construction and transformation

The coding sequences of *PtSnRK2* genes were cloned into the Gateway entry vector pENTR/D-TOPO or pCR8/GW/TOPO (Invitrogen), as described by Song et al. (2015), and were transferred to the destination vector pH35GS (Kubo et al., 2005) by the LR reaction using LR clonase II (Invitrogen). In the resulting plasmids, the *PtSnRK2* cDNA was expressed under the control of the cauliflower mosaic virus (CaMV) 35S promoter. The plasmids were electroporated into the *Agrobacterium tumefaciens* strain GV3101 (pMP90). A simplified version of the floral dip method was used for the transformation of Arabidopsis plants (Clough and Bent, 1998).

### Establishment of transgenic lines

For the screening of transgenic lines carrying the empty vector (vector controls) and *35S::PtSnRK2* (*PtSnRK2* overexpressors), T_1_ seedlings were grown in germination Murashige and Skoog (MS) medium containing 50 μg mL^−1^ hygromycin for 2 weeks, and the positive plants were transferred to soil for further growth. Although we failed to generate overexpressors of *PtSnRK2.2, PtSnRK2.6*, and *PtSnRK2.8*, more than 16 independent T_2_ lines were established for every other *PtSnRK2* genes.

To evaluate the expression levels of the introduced *PtSnRK2*, total RNAs were prepared from 7-day-old seedlings of the wild type, vector controls, and *PtSnRK2* overexpressors at the T_2_ generation using the RNeasy Mini kit (Qiagen). The first-strand cDNAs were synthesized using SuperScript III (Invitrogen) and subjected to RT-PCR analysis. The RT reaction was performed on a 20-μL scale, with 1 μL first-strand cDNA as a template for PCR, along with 0.5 μM of each gene-specific primer (see Table S2) and Ex Taq polymerase (TaKaRa). The PCR conditions were as follows: for the internal control gene *Ubp10*, 95°C for 5 min, followed by 35 cycles of 94°C for 20 s, 60°C for 30 s, and 72°C for 15 s, followed by 72°C for 7 min; for *PtSnRK2* genes (target genes), 95°C for 5 min, followed by 35 cycles of 94°C for 20 s, 60°C for 30 s, and 72°C for 1 min 15 s, and followed by 72°C for 7 min. The amplified PCR products were electrophoresed in 3 and 2% (w/v) agarose gels for the *Ubp10* and *PtSnRK2* genes, respectively, and gel images were analyzed using the AE-9020 E-shot II (ATTO; Figure S1). Information about the primer sets is provided in Table S11.

### Salt stress treatment

It is generally known that the expression level of *35S* promoter-driven genes would be decreased in T_3_ homozygous lines, so we decided to use T_2_ lines that were confirmed to show high expression levels of inserted *PtSnRK* genes as described above, for the salt stress treatment. Wild-type and transgenic T_2_ plants were grown at 22°C under LD conditions (16 h light/8 h dark) on 1/2-strength MS medium, after incubation at 4°C for 3 days. Then, 7-day-old plants were transferred to 1/2-MS medium plates with or without NaCl. For the survival rates and chlorophyll contents, 200 mM NaCl was included in the medium, and for the root growth phenotype, 100 mM NaCl was used. After an additional 4-day incubation, the numbers of surviving seedlings were counted to obtain survival rates (*n* = 20), and chlorophyll contents were measured as described below (*n* = 10). The primary root length was measured before and after a 5-day incubation on the NaCl plate, to calculate primary root elongation during salt stress treatment (*n* = 10). The treatments were repeated three times for survival rates and root length and six times for chlorophyll contents.

### Chlorophyll quantitation

Ten seedlings treated with 200 mM NaCl for 4 days were sampled in 3 mL (*N,N*-dimethylformamide, DMF). After incubation in DMF overnight at 4°C in the dark, A_646.8_, A_663.8_, and A_750_ were measured using the iMark Microplate Absorbance Reader (Bio-Rad). The chlorophyll quantitation was calculated by the formula:

[8.05 × (A_663.8_-A_750_) + 19.43 × (A_646.8_-A_750_) (μM) / the quantity of seedlings (mg)] (μM/mg) (Porra et al., 1989).

### *In silico* prediction of the three-dimensional structures of PtSnRK2 proteins

The amino acid sequences of PtSnRK2.5, PtSnRK2.7, and PtSnRK2.9 were submitted to the web-based SWISS-MODEL service (http://swissmodel.expasy.org/workspace/; Arnold et al., 2006), to build protein structure homology models using information on the crystal structure of the recombinant AtSnRK2.6 protein as a template (Ng et al., 2011).

### Microarray analysis

Seedlings of the wild-type and *PtSnRK2.7* overexpressor line 20 treated with or without 200 mM NaCl for 2 days were sampled for total RNA extraction. Microarray analysis was performed using ATH1 GeneChips (Affymetrix) according to the manufacturer's instructions on three independent biological replicates. Subsequent procedures of quality control, statistical analysis, and filtering were carried out using GeneSpring GX software (ver. 13.1; Agilent Technologies). Then, *p*-values were calculated for each probe using Welch's *t*-test (n = 3) for differences between the treated seedlings and the control seedlings, as well as between the wild-type and *PtSnRK2.7* overexpressor incubated without salt treatment. We used the Benjamin-Hochberg FDR method to control for false positives. A *p*-value cut-off of 0.01 was used to select genes whose expression changed with salt treatment. Fold-change values were also computed using GeneSpring GX, and we targeted those probes in which the change was upregulated or downregulated by more than 3-fold. Microarray data presented in this study were submitted to NCBI GEO (www.ncbi.nlm.nih.gov/geo/) and can be retrieved via accession number GSE79997.

### Gene ontology (GO) term analysis

Gene ontology (GO) term analysis was performed using the PANTHER classification system (Overrepresentation Test, release 20150430; Mi et al., 2013).

### Quantitative RT-PCR analysis

To evaluate the enhanced upregulation of salt stress response genes in *PtSnRK2.7* overexpressors, quantitative RT-PCR analysis was performed. Total RNAs were isolated from the seedlings treated with or without 200 mM NaCl for 2 days using Plant RNA Isolation Reagent (Invitrogen) and then purified using the RNeasy Mini Kit (QIAGEN). The first-strand cDNAs were synthesized as described above, and aliquots of the cDNA solution (0.5 μL for each gene) were used as templates for subsequent PCR amplification. The quantitative PCR analysis was performed using the LightCycler 480 System II (Roche) and LightCycler 480 SYBR Green I Master reagents (Roche). As an internal control, the *Ubc9* gene was used. Information on the primer sets is provided in Table S11.

## Results and discussion

### Overexpression of *PtSnRK2.5* and *PtSnRK2.7* improved salt stress tolerance in Arabidopsis

In the genome of *P. trichocarpa* (poplar), 12 PtSnRK2 genes have been identified (Song et al., 2015). For molecular functional analysis of PtSnRK2, we generated transgenic plants of *A. thaliana* (Arabidopsis) carrying the chimeric gene *35S::PtSnRK2*, in which the cDNA regions of *PtSnRK2* genes were regulated by the CaMV 35S promoter sequence, to overexpress poplar *PtSnRK2* genes in Arabidopsis. Unfortunately we could not obtain transgenic plants for *PtSnRK2.2, PtSnRK2.6*, and *PtSnRK2.8* overexpression; however, overexpressors of the other nine *PtSnRK2* genes were established successfully. It has been reported that overexpression of SnRK2 genes enhances abiotic stress tolerance (Umezawa et al., 2004; Diédhiou et al., 2008; Mao et al., 2010; Zhang et al., 2010, 2011; Tian et al., 2013). Thus, we examined salt stress tolerance in the *PtSnRK2* overexpressors.

First, 7-day-old seedlings from 16 independent T_2_ lines for each *PtSnRK2* overexpressor were transferred to medium containing 200 mM NaCl, and incubated for 4 days. The *prr9-11 prr7-10 prr5-10* triple mutant (d975) plant, which showed high salinity tolerance because of high expression of salt stress response genes (Nakamichi et al., 2009), was used as a positive control (Figure S2). The survival rates of seedlings after salt treatment demonstrated that the overexpressors of *PtSnRK2.5* and *PtSnRK2.7* showed relatively high survival rates among the *PtSnRK2* overexpressors. These two genes encode the subclass 2 PtSnRK2 proteins (Song et al., 2015). We further performed a detailed analysis of salt stress tolerance in the *PtSnRK2.5* and *PtSnRK2.7* overexpressors, using the wild-type and vector control plants as negative controls (**Figures 2**, **3**). For the *PtSnRK2.5* and *PtSnRK2.7* overexpressors, three independent lines were selected based on the expression levels of the introduced *PtSnRK2* genes (Figure S1). In the case of the negative controls, the seedling survival rates after 200 mM NaCl treatment were less than 20%, and the living seedlings exhibited yellowed leaves (**Figure 2**). In contrast, the *PtSnRK2.5* and *PtSnRK2.7* transgenic seedlings showed significantly higher survival rates (~55%) than those of the controls (**Figure 2**). All three independent *PtSnRK2.7* lines showed increased survival rates, whereas the *PtSnRK2.5* line 6 did not show a significantly enhanced survival rate (**Figure 2B**).

Next, the chlorophyll contents of 10 seedlings treated with salt stress were analyzed. The salt treatment greatly decreased chlorophyll contents in all plants; however, the chlorophyll contents of *PtSnRK2.5* lines 16 and 20, and all of the *PtSnRK2.7* lines were significantly higher than those of the wild-type and vector control (**Figure 2C**), in accordance with the survival rates. These results suggested that salt stress tolerance was more stable in the *PtSnRK2.7* overexpressors. Moreover, it was notable that although the chlorophyll contents in the absence of salt stress were at almost the same levels among the wild-type, vector control, and *PtSnRK2.7* lines, but the *PtSnRK2.5* lines 16 and 20 showed lower amounts of chlorophyll under normal growth conditions (**Figure 2C**). Thus, *PtSnRK2.5* overexpression may have affected chlorophyll biosynthesis continuously.

In addition to the increased survival rates and chlorophyll contents, the living seedlings of the *PtSnRK2.5* and *PtSnRK2.7* transgenic lines seemed to be larger than those of the negative controls under salt stress conditions (**Figure 2A**). To clarify the effects of *PtSnRK2* overexpression on seedling growth, we checked the growth of primary roots after salt treatment. Because the 200 mM NaCl treatment almost completely inhibited primary root elongation in both the overexpressors and negative controls, we used 100 mM NaCl conditions to observe primary root elongation. Our data demonstrated no difference in primary root elongation in the mock-treated seedlings (**Figure 3**), indicating that these two *PtSnRK2* genes are not involved in root elongation regulation, unlike the Arabidopsis subclass II *AtSRK2C/SnRK2.8* gene, overexpression of which was reported to enhance seedling root growth (Shin et al., 2007). However, after a 5-day incubation with 100 mM NaCl, the primary roots of *PtSnRK2.5* line 20 and all of the *PtSnRK2.7* lines elongated significantly more than did those of the negative controls; the negative controls showed elongation of less than 20 mm, whereas the elongated lengths of *PtSnRK2.5* line 20 and all of the *PtSnRK2.7* lines were ~25 mm (*p* < 0.01, *t*-test). These observations indicated that overexpression of *PtSnRK2.7*, and possibly also *PtSnRK2.5*, can suppress the inhibition of root elongation by high salinity stress.

**Figure 1:**
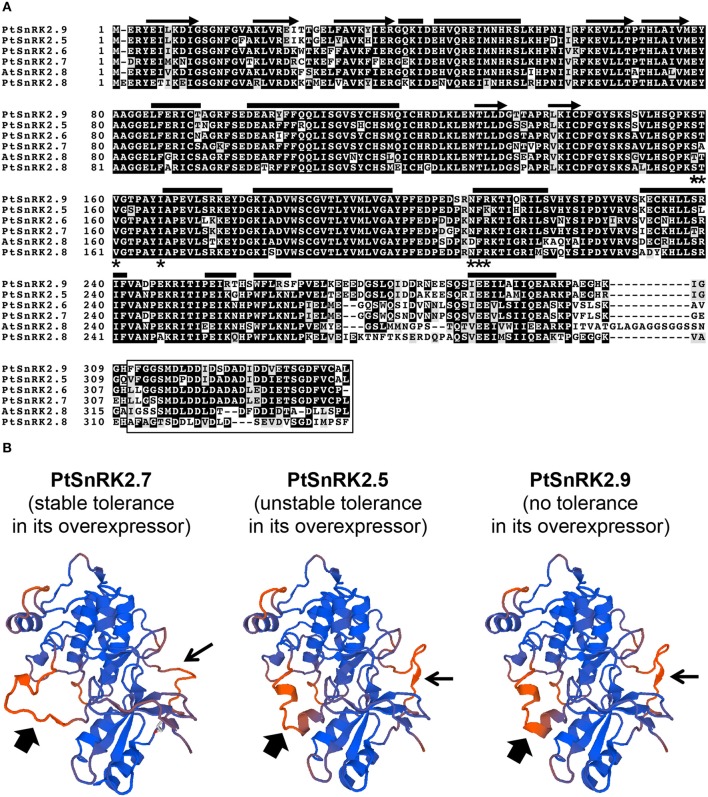
**SnRK2 proteins of *Populus trichocarpa* (poplar, Pt), and *Arabidopsis thaliana* (Arabidopsis, At)**. **(A)** Amino acid sequence alignment of subclass 2 PtSnRK2 proteins with AtSnRK2.8. The residues putatively corresponding to the α-helix, β-sheet, PP2C-interaction residues, and the entire ABA box are marked by bars, arrows, asterisks, and the box, respectively. **(B)** *In silico* modeling of the three-dimensional structures of PtSnRK2.5, PtSnRK2.7, and PtSnRK2.9 proteins. Homology modeling was performed based on the crystal structure of AtSnRK2.6 using the web-based SWISS-MODEL service (http://swissmodel.expasy.org/workspace/; Arnold et al., 2006). Thick and narrow arrows indicate regions presumed to differ in their three-dimensional architecture among the PtSnRK2 proteins because of differences in amino acid sequences.

**Figure 2:**
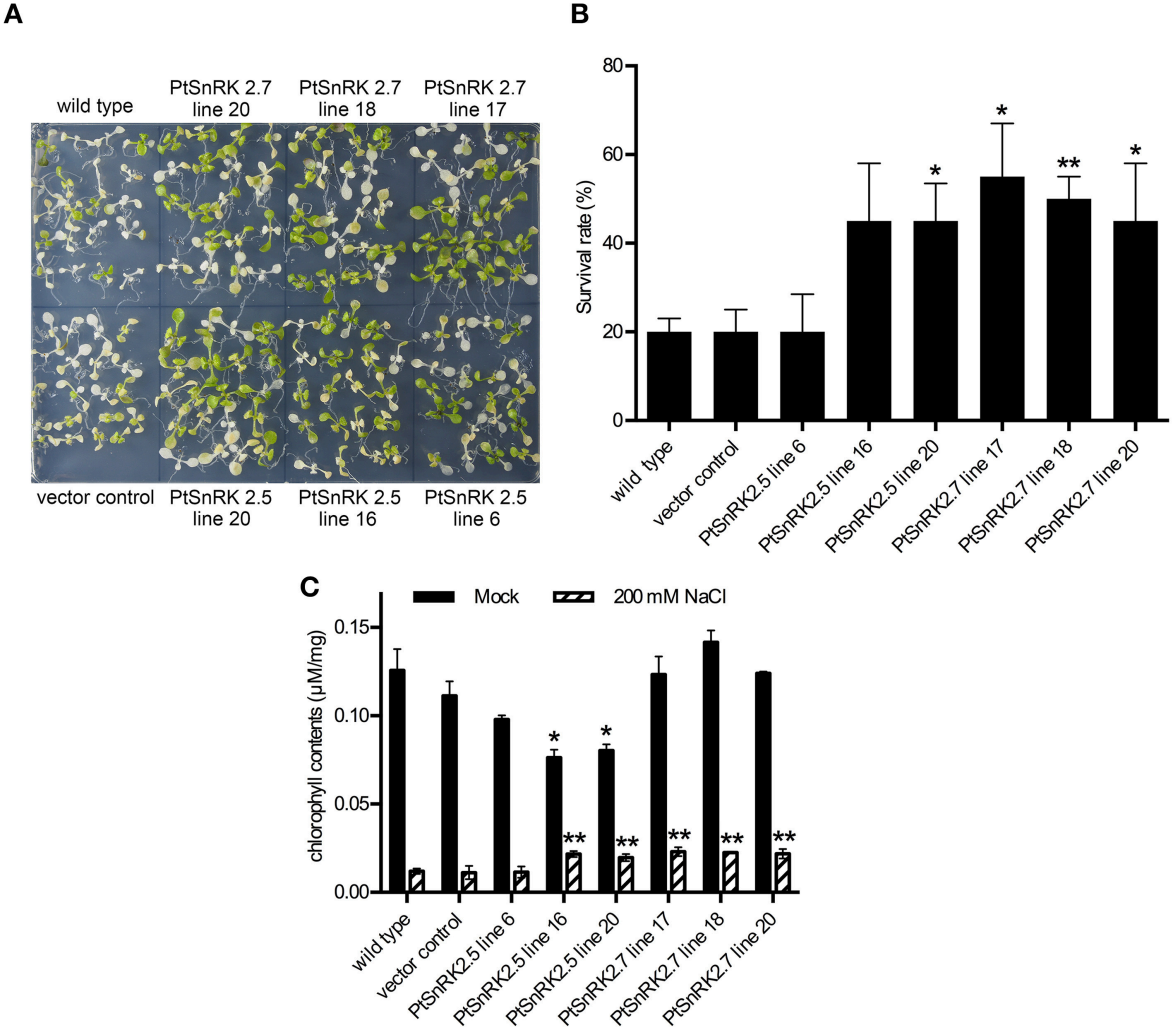
**High salinity tolerance phenotype of transgenic Arabidopsis overexpressing *PtSnRK2.5* and *PtSnRK7***. **(A)** Seedlings of the wild-type, vector control, and overexpressors of *PtSnRK2.5* and *PtSnRK2.7* treated with 200 mM NaCl for 4 days. **(B)** Survival rates determined by observations after a 4-day salt stress treatment. The green seedlings were counted as living seedlings, and the percentages of live seedlings were calculated using 20 seedlings for each line. **(C)** Chlorophyll contents determined from 20 seedlings after a 4-day salt stress treatment. Results are means ± SE (*n* = 3). Asterisks indicate statistically significant differences between transgenic and wild-type plants (Student's *t*-test; ^*^p < 0.05; ^**^p < 0.01).

**Figure 3:**
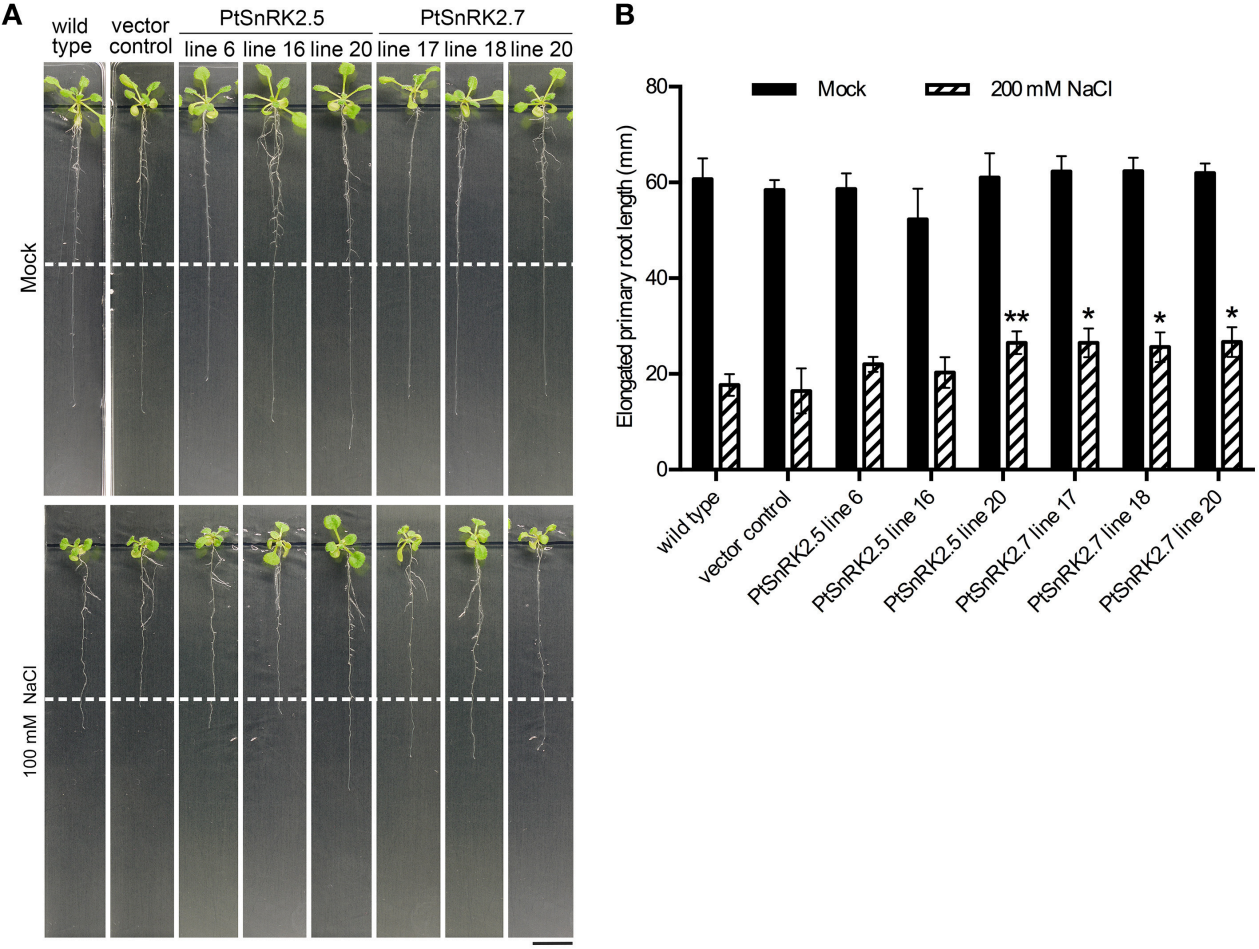
**Primary root elongation of transgenic Arabidopsis overexpressing *PtSnRK2.5* and *PtSnRK2.7* during salt treatment**. **(A)** Seedlings of the wild-type, vector control, and overexpressors of *PtSnRK2.5* and *PtSnRK2.7* treated with 100 mM NaCl for 5 days. The positions of the edges of the root tips before salt treatment are indicated by the white dotted lines. Bar = 20 mm. **(B)** Increase in root length after salt stress treatment. Results are means ± SE (*n* = 15). Asterisks indicate statistically significant differences between transgenic plants and the wild-type (Student's *t*-test; ^*^p < 0.05; ^**^p < 0.01).

Our data on the *PtSnRK2.5* and *PtSnRK2.7* transgenic lines demonstrated different phenotypic characteristics between the *PtSnRK2.5* and *PtSnRK2.7* lines. Thus, it was suspected that the molecular basis for the enhancement of salt stress tolerance would be different between the *PtSnRK2.5* and *PtSnRK2.7* transgenic lines. In particular, the *PtSnRK2.5* lines showed unstable salt stress tolerance (**Figures 2**, **3**) and defects in chlorophyll content even under normal growth condition (**Figure 2C**). The subclass 2 PtSnRK2 proteins have highly similar amino acid sequences (Figure 1A), and the amino acid sequences of PtSnRK2.5 (unstable, but significantly enhanced salinity tolerance), PtSnRK2.7 (stable and high tolerance), and PtSnRK2.9 (no obviously increased tolerance) did not show major differences between them (Figure 1A). *In silico* modeling of the three-dimensional structures of these proteins, based on the crystal structure of AtSnRK2.6 (Ng et al., 2011), revealed that some of the differences in amino acid sequences among these PtSnRK2 proteins could correspond to the molecular surface regions of these proteins (Figure 1B), possibly leading to changes in the molecular activities of the SnRK2 proteins. The different SnRK2 protein activities due to subtle substitutions of amino acids could result in the different strengths of salt stress tolerance among the overexpressors.

### *PtSnRK2.7* overexpression widely and largely influenced the transcriptome in response to salt stress

**Figure 4:**
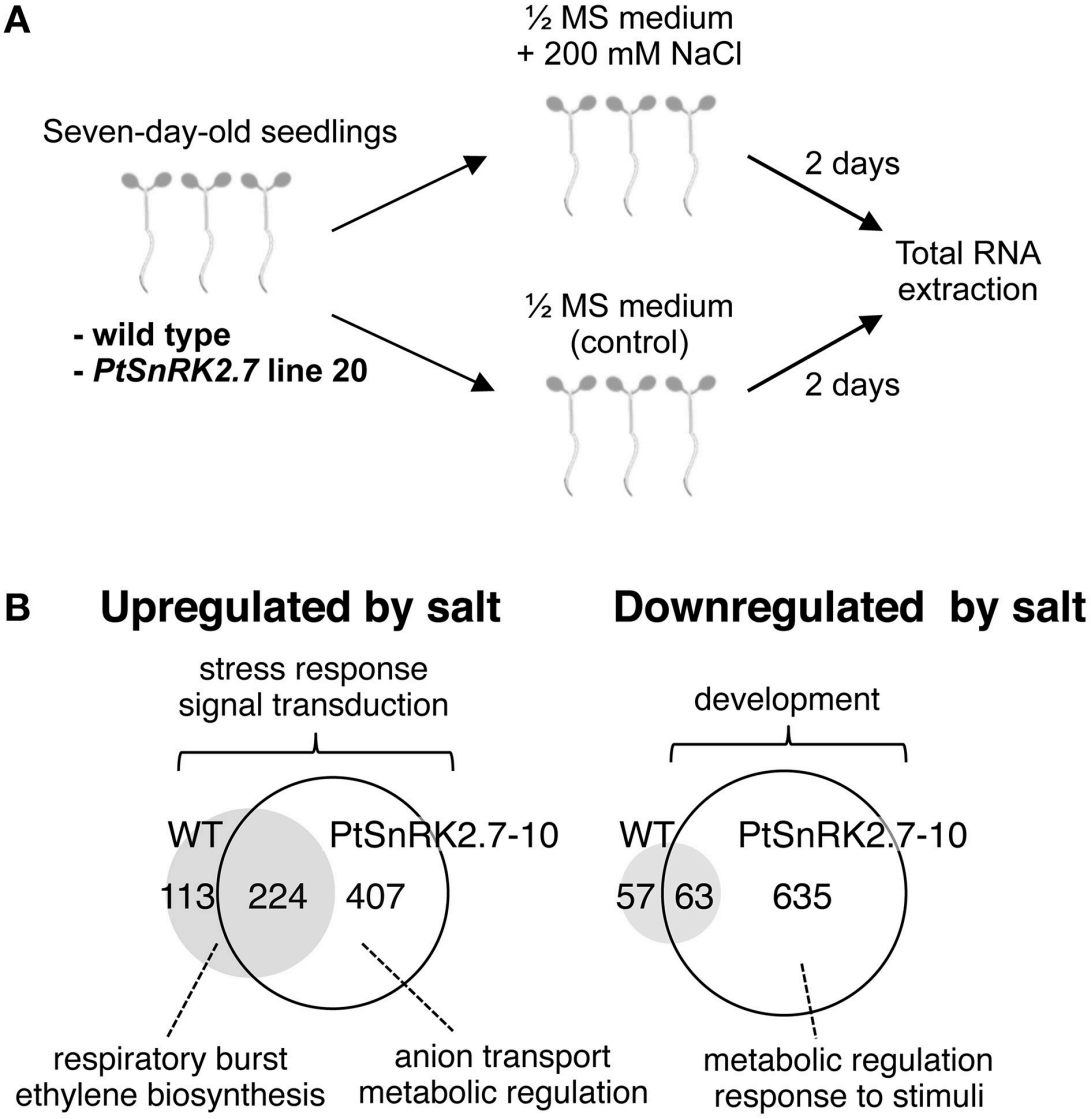
**Microarray analysis of the wild-type and PtSnRK2.7 overexpressor line 20**. **(A)** Overview of sample preparation for microarray analysis. **(B)** Venn diagram of upregulated and downregulated genes after salt stress treatment in the wild-type and PtSnRK2.7 overexpressor.

We next performed transcriptomic analyses of the wild-type and overexpressors during salt stress treatment. Based on the data described above (Figures 2, 3), we selected *PtSnRK2.7* line 20, which showed stable and strong salt stress tolerance among the transgenic lines, for the gene chip analysis. Then, 7-day-old seedlings of the wild-type and *PtSnRK2.7* line 20 were treated with or without 200 mM NaCl for 2 days and then sampled to extract total RNA. The extracted total RNA samples were subjected to microarray analysis using Affymetrix ATH1 GeneChips (Figure 4A). First, we compared transcriptomic data between the wild-type and *PtSnRK2.7* overexpressor, which were mock-treated, to examine the effects of *PtSnRK2.7* overexpression on gene expression under normal growth conditions. Thirty and 79 genes were shown to be upregulated and downregulated in the *PtSnRK2.7* overexpressor, respectively (FC > 2, *p* < 0.05; Table S1). It has been reported that in AtSRK2C/SnRK2.6 and TaSnRK2.8 overexpressors, stress response-related genes including RD29A and DREB1A/CBF3, and ABA biosynthetic genes are upregulated continuously (Umezawa et al., 2004; Zhang et al., 2011). However, these genes were not changed by *PtSnRK2.7* overexpression (Table S1). The gene ontology (GO) term analysis revealed that stress-related metabolic genes, including lipid metabolism and flavonoid metabolism, were significantly downregulated in the *PtSnRK2.7* overexpressor (Table 1). These results suggest that *PtSnRK2.7* overexpression could continuously affect specific ranges of gene expression regulation, which do not overlap with the primary targets of AtSRK2C/SnRK2.6 and TaSnRK2.8 in Arabidopsis.

**Table 1:** **GO term analysis of the differentially expressed genes between the wild-type and *PtSnRK2.7* overexpressor grown in the absence of salt stress treatment**.

**GO biological process complete**	**Fold Enrichment**	***p*-value**
Anthocyanin-containing compound biosynthetic process	44.44	4.52E-03
Anthocyanin-containing compound metabolic process	40.00	7.29E-03
Cellular response to phosphate starvation	33.33	1.12E-09
Galactolipid biosynthetic process	33.33	2.61E-07
Galactolipid metabolic process	33.33	2.61E-07
Glycolipid biosynthetic process	28.57	1.06E-06
Flavonoid biosynthetic process	26.09	2.69E-04
Flavonoid metabolic process	24.00	4.11E-04
Liposaccharide metabolic process	23.53	4.26E-06
Glycolipid metabolic process	23.53	4.26E-06
Membrane lipid biosynthetic process	22.22	6.69E-06
Response to UV-B	20.83	9.49E-03
Cellular response to starvation	18.18	6.92E-09
Cellular response to nutrient levels	17.65	8.89E-09
Membrane lipid metabolic process	17.39	4.15E-05
Response to starvation	17.39	1.08E-08
Cellular response to extracellular stimulus	16.88	1.88E-09
Cellular response to external stimulus	16.67	2.26E-09
Response to nutrient levels	16.67	1.82E-08
Response to extracellular stimulus	16.05	3.73E-09
Pigment biosynthetic process	13.73	1.65E-03
Pigment metabolic process	10.94	7.65E-03
Negative regulation of transcription, DNA-templated	8.43	3.85E-02
Negative regulation of RNA biosynthetic process	8.43	3.94E-02
Negative regulation of nucleic acid-templated transcription	8.43	3.94E-02
Negative regulation of RNA metabolic process	8.43	4.03E-02
Cellular response to stress	5.62	1.09E-04
Response to external stimulus	3.65	2.57E-03
Single-organism biosynthetic process	2.99	6.83E-03

To examine the impact of *PtSnRK2.7* overexpression on the salt stress response, the genes upregulated or downregulated by salt treatment were compared between the wild-type and *PtSnRK2.7* overexpressor. In the wild-type, 337 and 120 genes were upregulated and downregulated by salt treatment, respectively (FC > 3, *p* < 0.01; Figure 4B and Tables S2, S3). Notably, the *PtSnRK2.7* overexpressor showed greater numbers of genes whose expression was changed by salt treatment compared with the wild-type; 631 and 698 genes were upregulated and downregulated in the *PtSnRK2.7* overexpressor, respectively (FC > 3, *p* < 0.01; Figure 4B and Tables S4, S5), suggesting that *PtSnRK2.7* overexpression affected the expression of a wide range of genes during salt stress responses in Arabidopsis. The GO analysis indicated that the genes functioning in stress responses, including the signaling pathway of stress-related phytohormones (ABA, ethylene, jasmonic acid, and salicylic acid) and in signal transduction by protein phosphorylation, were commonly upregulated between the wild-type and *PtSnRK2.7* overexpressor (Figure 4B and Tables S6–S8). Additionally, developmental process-related genes were commonly downregulated in the wild-type and *PtSnRK2.7* overexpressor (Figure 4B and Table S10).

We also found that some GO terms were enriched in the upregulated genes in genotype-dependent manners, such as “respiratory burst” and “ethylene biosynthetic process” found only in the wild-type (Figure 4B and Table S6) and “purine nucleoside transmembrane transport,” “amino acid transport,” “anion transmembrane transport,” and “nucleic acid metabolic process” found only in the *PtSnRK2.7* overexpressor (Figure 4B and Table S7). The overrepresentation of the GO terms related to “anion transmembrane transport” in the *PtSnRK2.7* overexpressor would suggest the enhancement of ion homeostasis activity as a result of *PtSnRK2.7* overexpression, possibly leading to higher salt stress tolerance. Our results also showed wild-type-specific enrichment of the term “ethylene biosynthetic process” in upregulated genes (Figure 4B and Table S6). It has been reported that crosstalk between ABA and ethylene is a critical factor in determining salt stress tolerance in Arabidopsis (Dong et al., 2011); thus, *PtSnRK2.7* overexpression may influence the phytohormonal modulating system of abiotic stress responses. Moreover, in the *PtSnRK2.7* overexpressor, more than 5-fold more genes were downregulated by salt treatment than those in the wild type. These downregulated genes are involved in a wide range of molecular functions, including metabolic regulation and responses to stimuli (Figure 4B and Table S9), indicating that the reason for the high salt stress tolerance in the *PtSnRK2.7* overexpressor may be, at least partially, attributed to its significant impact on metabolic regulation.

**Figure 5:**
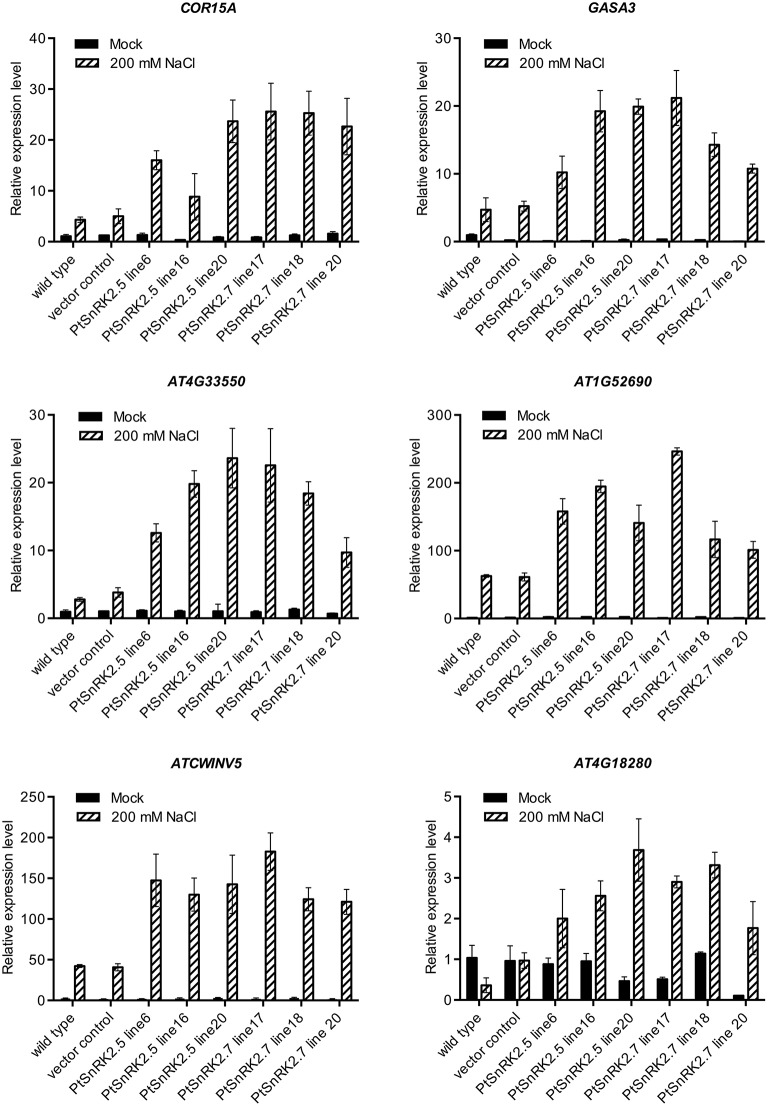
**Quantitative RT-PCR analysis of transcript levels of COR15A, GASA3, At4G33550, AtCWINV5, *At1G52690*, and *At4G18280* in the wild-type, vector control, and overexpressors of *PtSnRK2.5* and *PtSnRK2.7***. Seedlings treated with or without 200 mM NaCl for 2 days were analyzed. Results are means ± SE (*n* = 3).

Comparison of transcriptomic data also suggested that the altered expression levels of upregulated genes were greater in the *PtSnRK2.7* overexpressor than in the wild-type (Tables S2, S4). To confirm this, we selected six genes from the functional categories of stress response (*COR15A*, Artus et al., 1996), phytohormonal signaling (*GASA3*, Herzog et al., 1995), cell wall-related proteins (*AT1G52690* and *AtCWINV5*, Sherson et al., 2003), and lipid metabolism (*AT4G33550*), based on the transcriptomic data, and subjected them to quantitative RT-PCR analysis. *AT4G18280*, which was upregulated only in the *PtSnRK2.7* overexpressor, was also tested to evaluate the consistency of the results between the gene chip analysis and quantitative RT-PCR analysis. Indeed, *AT4G18280* expression was not induced by salt stress in the wild-type or vector control, whereas it was highly upregulated in the *PtSnRK2.7* line 20 in accordance to the results of gene chip analysis (Figure 5 and Table S4). The results showed that the tested genes were actively upregulated by salt treatment in the *PtSnRK2.7* overexpressors compared with the wild-type and vector control (Figure 5). Our data also demonstrated that *PtSnRK2.5* overexpression had similar effects on the expression of these genes. Thus, it is supposed that *PtSnRK2.5* overexpression affected the transcriptome in a similar fashion to *PtSnRK2.7* overexpression.

It has been reported that the overexpression of Arabidopsis *AtSnRK2.8* enhanced the stress tolerance of Arabidopsis, probably through the continuous upregulation of key genes for stress responses, such as *RD29A, COR15A, AtGolS3, DREB1A*, and *PKS18* (Umezawa et al., 2004). However, our results showed that overexpression of poplar *SnRK2* genes did not constitutively induce these well-known stress responsive genes. Rather, the high salt stress tolerance of the *PtSnRK2* overexpressors may be explained by changes in transcriptomic regulation for a wide range of metabolic regulatory genes (Figures 4, 5; Table 1 and Tables S1–S10). It is notable that the genes related to anion transport activity were upregulated in the *PtSnRK2.7* overexpressor specifically, because similar effects on ion homeostasis-related genes were reported in the case of overexpression of rice *SAPK4* (Diédhiou et al., 2008). Moreover, a comparative transcriptomic analysis using salt-tolerant and non-tolerant species of poplar indicated that prominent factors for high salt tolerance were not overexpression of the stress responsive pathway, but rather enhanced activities for osmotic adjustment, ion compartmentalization, and detoxification of reactive oxygen species in poplar (Chen and Polle, 2010). Thus, our results may reflect differences in the regulatory targets of SnRK2 proteins between AtSnRK2 and PtSnRK2, which could be related to diversified molecular strategies of stress adaption in each plant species, as suggested by Zhang et al. (2014). Future comparative analyses on the mode of actions of SnRK2 proteins derived from different plant species may provide important information on novel strategies to improve stress tolerance of crops and other useful plants.

## Author contributions

XS designed and performed the experiments, and drafted the manuscript. MO designed the experiments, analyzed the data, and wrote the manuscript. XY and CH contributed to the data analysis. QZ and TD drafted the manuscript. All authors contributed to and approved the final manuscript.

### Conflict of interest statement

The authors declare that the research was conducted in the absence of any commercial or financial relationships that could be construed as a potential conflict of interest.

## Supplementary material

Figure S1 **RT-PCR analysis of seedlings of the wild-type, vector control, and overexpressors of *PtSnRK2.5* and *PtSnRK2.7***.

Figure S2 **Primary screening of transgenic Arabidopsis overexpressing PtSnRK2 genes for the salt tolerance**. Seedlings of the d975 (positive control), vector control, and overexpressors of *PtSnRK2* genes were treated with 200 mM NaCl, and checked their survival rates after 3, 4, and 5 days of NaCl treatment. A part of results for the transgenic lines of *PtSnRK2.5* **(A)** and *PtSnRK2.9* **(B)** treated with 200 mM NaCl for 4 days were shown.

Table S1 **Differentially expressed genes between the wild type and PtSnRK2.7 overexpressor (FC>2, *p* < 0.05)**.

Table S2 **Upregulated 337 genes in the wild type by the salt treatment (FC>3, *p* < 0.01)**.

Table S3 **Downregulated 120 genes in the wild type by the salt treatment (FC>3, *p* < 0.01)**.

Table S4 **Upregulated 631 genes in the PtSnRK2.7 overexpressor by the salt treatment (FC>3, *p* < 0.01)**.

Table S5 **Downregulated 698 genes in the PtSnRK2.7 overexpressor by the salt treatment (FC>3, *p* < 0.01)**.

Table S6 **GO term analysis on wild-type-specific upregulated genes by salt treatment**.

Table S7 **GO term analysis on PrSnRK2.7 overexpressor-specific upregulated genes by salt treatment**.

Table S8 **GO term analysis on commonly upregulated genes between the wild type and PtSnRK2.7 overexpressor by salt treatment**.

Table S9 **GO term analysis on PrSnRK2.7 overexpressor-specific downregulated genes by salt treatment**.

Table S10 **GO term analysis on commonly downregulated genes between the wild type and PtSnRK2.7 overexpressor by salt treatment**.

Table S11 **Oligonucleotides used in this paper**.

## Abbreviations

SnRK2: SNF1-related protein kinases 2
ABA: abscisic acid.
