# Supplementary figures and images for: Heterologous Overexpression of Poplar SnRK2 Genes Enhanced Salt Stress Tolerance in Arabidopsis thaliana
Source: Front Plant Sci. 2016 May 9;7:612. doi: 10.3389/fpls.2016.00612 (PMC4860416; doi:10.3389/fpls.2016.00612)

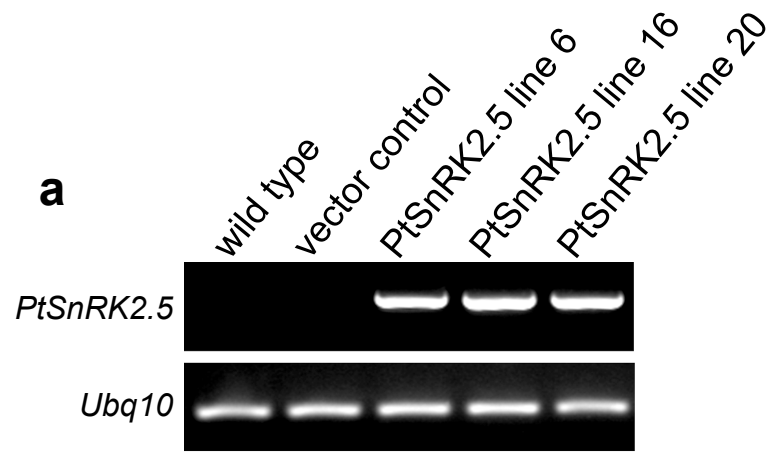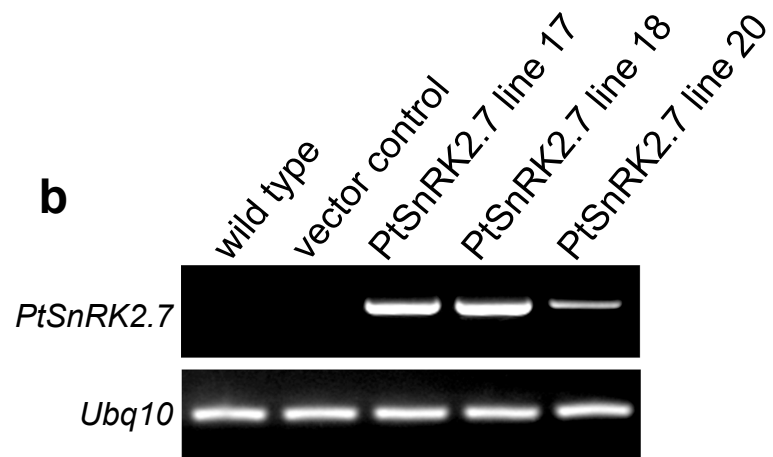

Supplement: Figure S1 — RT-PCR analysis of seedlings of the wild-type, vector control, and overexpressors of PtSnRK2.5 and PtSnRK2.7. [file Image1.PDF]

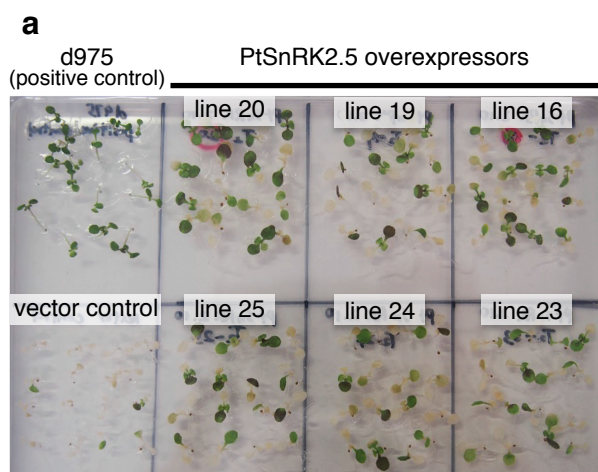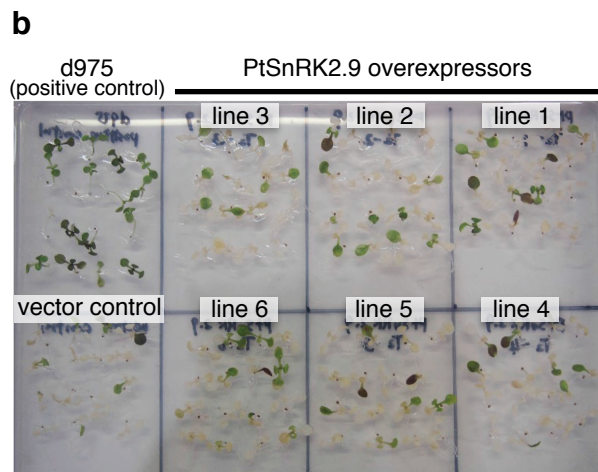

Supplement: Figure S2 — Primary screening of transgenic Arabidopsis overexpressing PtSnRK2 genes for the salt tolerance. Seedlings of the d975 (positive control), vector control, and overexpressors of PtSnRK2 genes were treated with 200 mM NaCl, and checked their survival rates after 3, 4, and 5 days of NaCl treatment. A part of results for the transgenic lines of PtSnRK2.5 (A) and PtSnRK2.9 (B) treated with 200 mM NaCl for 4 days were shown. [file Image2.PDF]
